# Complete plastid genome of *Gentiana leucomelaena* Maxim. (Gentianaceae) and phylogenetic analysis

**DOI:** 10.1080/23802359.2021.1972872

**Published:** 2021-09-15

**Authors:** Hui-Yuan Ya, Yu-Wei Miao

**Affiliations:** a School of Food and Drug, Luoyang Normal University, Luoyang, People’s Republic of China; b Construction Management Committee of Xining Garden Expo Park and XiBu Forest Park, Xining, People’s Republic of China

**Keywords:** *Gentiana leucomelaena*, Gentianaceae, phylogenetic relationship, plastome

## Abstract

The complete plastid genome of *Gentiana leucomelaena* Maxim., belonging to the most species-rich section *Chondrophyllae* in *Gentiana*, was determined and analyzed in this study. It has a circular-mapping molecular with the length of 131,856 bp, the shortest one among all available *Gentiana* plastomes. *Gentiana leucomelaena* has gene mutation, for example *ndh* and *rpl*2 intron, and reversed SSC region comparing with the available species in sections *Cruciata*, *Frigida*, *Kudoa*, *Isomeria* and *Microsperma*. Phylogenetic analysis showed that *G. leucomelaena* clustered together with section *Cruciata* with a long branch. The plastome provides in this work will contribute to elucidate the phylogenetics and evolution in *Gentiana*.

*Gentiana* is an alpine group containing 362 species (Ho and Liu 2001), with the Qinghai-Tibet Plateau as the distribution and diversity center (Ho and Liu 2001; Favre et al., 2016). Among the 13 sections in *Gentiana*, section *Chondrophyllae* Bunge is the largest one containing about 180 species which are further divided into 12 series (Ho and Liu 2001; Favre et al. 2020). *Gentiana leucomelaena* Maxim., belonging to section *Chondrophyllae* series *Humiles* Marquand, has distribution range in Qinghai-Tibetan Plateau and adjacent areas. As the most species-rich section, there is very limited available plastome data in section *Chondrophyllae* at present (Fu et al. 2021).

Herein, we reported and characterized the complete plastome of *G. leucomelaena* (MT905404). One *G. leucomelaena* individual was collected from Jiangda, Tibet, China (31°38′N, 98°25′E). The voucher specimen was deposited at Herbarium of Luoyang Normal University (Bing Cai, 987869364@qq.com) under the voucher number Miao1902. The whole plant was used for DNA extraction using a Dzup plant genomic DNA extraction kit (Sangon, Shanghai, China). The fragmented genomic DNA was sequenced using Illumina HiSeq 2500 (Novogene, Tianjing, China), yielding 3 Gb of 150-bp paired-end reads. The plastome was *de novo* assembled by NOVOPlasty 2.6.1 (Dierckxsens et al. 2016) and annotated by GeSeq (Tillich et al. 2017) using the default parameters. Comparative analysis was conducted by mVISTA (Frazer et al. 2004) with species from five plastome-available *Gentiana* sections, *Cruciata* (Zhou et al. 2018), *Frigida* (She et al. 2019; Sun, Wang, et al. 2019), *Kudoa* and *Isomeria* (Fu et al. 2016; Sun et al. 2018) and *Microsperma* (Sun, Zhou, et al. 2019). Shared protein coding genes in available *Gentiana* plastomes were extracted and aligned by using MAFFT (Katoh et al. 2002). Using concatenated protein coding genes, maximum likelihood phylogenetic analyses were conducted with IQ-TREE (Nguyen et al. 2015) in PhyloSuite (Zhang et al. 2020) with 1000 replicates. *Metagentiana rhodantha* (MN822304) was served as the outgroup.

The complete *G. leucomelaena* plastome is a circular-mapping molecule with the length of 131,856 bp, the shortest in all available *Gentiana* plastomes. The LSC, IR and SSC regions were 75,476, 23,259 and 9862 bp, respectively. A total of 122 genes were annotated, containing 80 protein-coding genes, 34 tRNA genes and 8 rRNA genes. Comparison analysis indicated that plastome of *G. leucomelaena* has numerous gene losses such as *ndh* complex and *rpl*2 intron, which is similar with *G*. section *Kudoa* (Sun et al. 2018). In addition, the SSC region in *G. leucomelaena* plastome is reversed comparing with all other *Gentiana* plasomtes.

Comparison analysis showed that the hotspots among sections *Chondrophyllae*, *Cruciata*, *Frigida*, *Isomeria*, *Kudoa* and *Microsperma* located at intergenic regions, for instance *trn*H-GUG–*psb*A, *atp*H–*atp*I, *pet*N–*trn*D, *rbc*L–*acc*D and *trn*L-UAG–*ccs*A. The phylogenetic relationships among studied sections were fully supported. Phylogenetic analysis showed that *G. leucomelaena* was sister to section *Cruciata* with a long branch (Figure 1), which was consistent with previous study (Favre et al. 2016). The determination of the *G. leucomelaena* plastome sequences provided new molecular data to illuminate the phylogenetics and molecular evolution of *Gentiana*.

**Figure 1.**
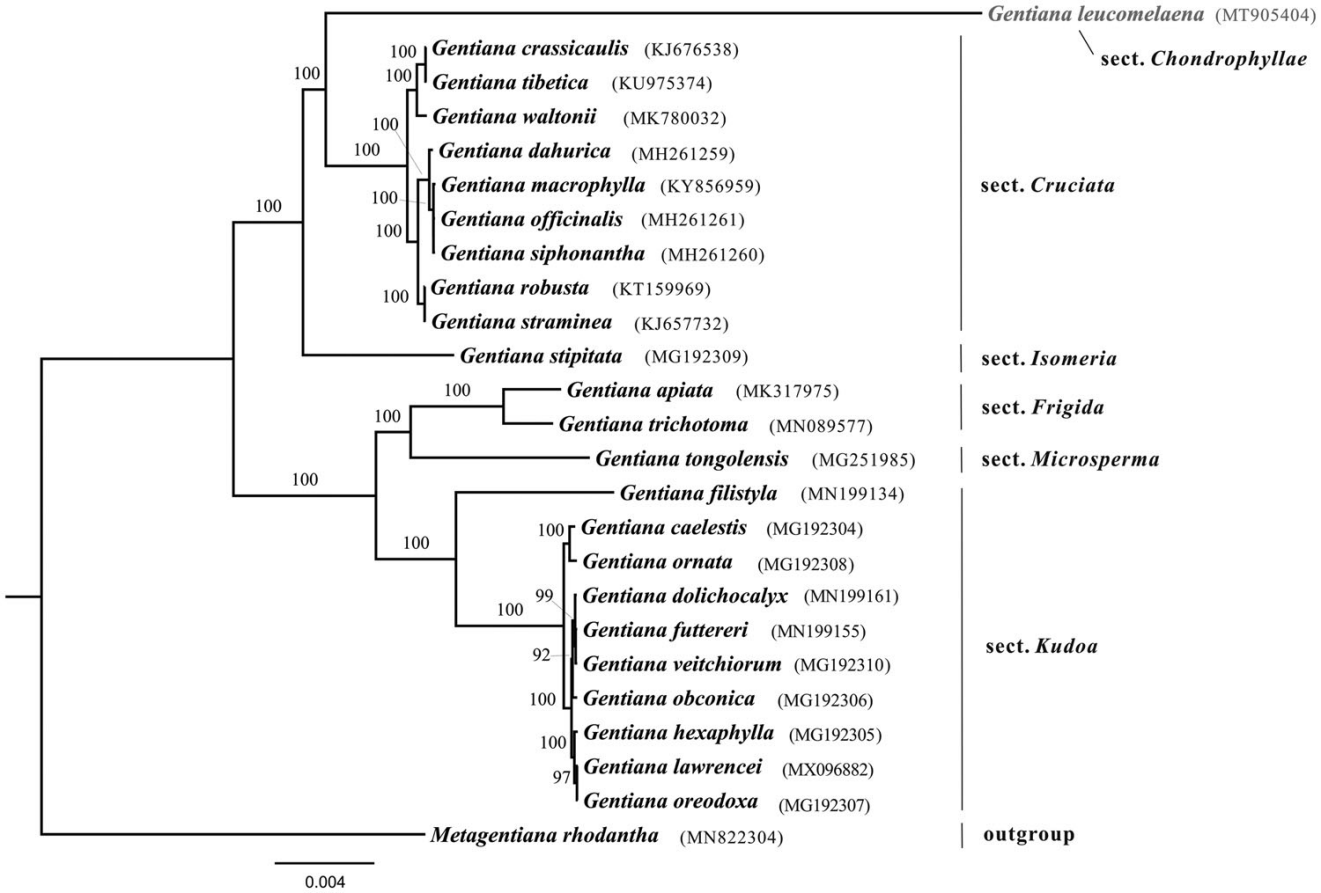
Maximal likelihood tree of *Gentiana* based on protein-coding genes in plastome. Numbers above the branches present bootstrap supports. The species name in gray indicates the newly sequenced in this study.

## Data Availability

The genome sequence data that support the findings of this study are openly available in GenBank of NCBI at (https://www.ncbi.nlm.nih.gov/) under the accession no. MT905404. The associated BioProject, SRA, and Bio-Sample numbers are PRJNA738973, SRR14859452, and SAMN19768589, respectively.
